# Higher-dimensional physical models with multimemory indices: analytic solution and convergence analysis

**DOI:** 10.1186/s13662-020-02822-7

**Published:** 2020-07-16

**Authors:** Imad Jaradat, Marwan Alquran, Ruwa Abdel-Muhsen, Shaher Momani, Dumitru Baleanu

**Affiliations:** 1 Department of Mathematics & Statistics, Jordan University of Science and Technology, Irbid, 22110 Jordan; 2 Department of Mathematics and Sciences, College of Humanities and Sciences, Ajman University, Ajman, UAE; 3 Department of Mathematics, Faculty of Science, The University of Jordan, Amman, 11942 Jordan; 4 Department of Mathematics, Cankaya University, 06530 Balgat, Ankara Turkey; 5 Institute of Space Sciences, Magurele, Bucharest, Romania; 6 Department of Medical Research, China Medical University, Taichung, Taiwan

**Keywords:** 26A33, 41A58, 35R11, 35C10, Memory index, Fractional PDEs, Analytic solution

## Abstract

The purpose of this work is to analytically simulate the mutual impact for the existence of both temporal and spatial Caputo fractional derivative parameters in higher-dimensional physical models. For this purpose, we employ the *γ̅*-Maclaurin series along with an amendment of the power series technique. To supplement our idea, we present the necessary convergence analysis regarding the *γ̅*-Maclaurin series. As for the application side, we solved versions of the higher-dimensional heat and wave models with spatial and temporal Caputo fractional derivatives in terms of a rapidly convergent *γ̅*-Maclaurin series. The method performed extremely well, and the projections of the obtained solutions into the integer space are compatible with solutions available in the literature. Finally, the graphical analysis showed a possibility that the Caputo fractional derivatives reflect some memory characteristics.

## Introduction

Fractional mathematical models have shown the ability to describe the dynamics of some natural phenomena and nonlocal systems that inherit memory properties [1–3]. They are ubiquitous in many areas such as physics, chemistry, biology, control theory, signal and image processing, and economics. For this reason, many of the existing nonlinear PDEs that describe different phenomena have been remodeled in the sense of fractional derivatives, and their chaotic behavior and solutions have been reported in many cases by using developed methods based on wavelets and B-spline collocation ideas [4–9], fractional power (M-)series [10–16], finite difference schemes [17–19], variational iteration methods [20, 21], and (q-)homotopy analysis approaches [22–25].

To mention a few recent works in fractional calculus, a series of interesting results appeared in the literature. For example, the existence and controllability have been investigated for fractional neutral functional (integro)differential equations with nonlocal conditions and with infinite delay in Banach spaces [26–30]. The dynamical structures and the epidemic prophecy have been studied for the novel coronavirus (2019-nCoV) with a nonlocal operator defined in the Caputo sense [31, 32]. The nondifferentiable behavior of heat conduction of the fractal temperature field in homogeneous media was shown in [33]. A fractional epidemiological model describing computer viruses with an arbitrary order derivative having a nonsingular kernel was analyzed in [34]. The dynamics of hepatitis B viral infection with DNA-containing capsids, the liver hepatocytes, and the humoral immune response via fractional differential mathematical models are presented and investigated in [35]. A new immunogenetic tumor model with nonsingular fractional derivative was studied in [36].

Various fractional derivative operators are proposed in the literature. The Riemann–Liouville and Caputo fractional operators are the most common approach of defining fractional derivatives. One common characteristic of these operators is the singularity of their kernels. Although these fractional derivative operators played a vital role in modeling several real-life phenomena, certain phenomena related to material heterogeneities cannot be well modeled in the sense of these fractional derivatives [37]. As a result, general fractional derivatives with nonsingular kernels in terms of the Mittag-Leffler, exponential, trigonometric, Bessel, and Rabotnov fractional-exponential functions were submitted and utilized in modeling many different real-life problems [37–45].

The central focus of almost all the proposed methods was mainly exploring the influence of the time-fractional derivative. However, several studies have revealed that the power-law memory instilled in process and materials could also be in the space coordinate [46, 47]. Motivated by this lack, some analytical methods were recently developed to handle and study mathematical models embedded entirely in a fractional space [48–51]. Continuing in this direction, this research examines the joint influence for the existence of both time and space fractional derivatives in higher-dimensional PDEs. To this end, we merged a new analytical solution representation endowed with multifractional derivative parameters together with the classical power series technique to find the solution of the mathematical models living entirely in a fractional space. Also, we graphically studied the behavior of the obtained solutions and noticed that these solutions converge homotopically, when the Caputo fractional derivatives move from zero to one, to the solution of the integer version of the problem. In some sense, this supports the idea that these fractional derivative parameters act as memory indices.

## Analytic solution ansatz of higher-dimensional FPDEs

Here we propose an analytic solution ansatz of higher-dimensional partial differential equations entirely living in the fractional space. Then we provide a theoretical frame of the solution ansatz convergence.

### Definition 2.1

A trivariate Maclaurin *γ̅*-series (abbreviated by *γ̅*-Maclaurin) is a rearrangement for a fractional Cauchy product series in the form

2.1 $$ \begin{aligned}[b] {\sum _{\substack{\ell_{1}+\ell_{2}+\ell_{3}=0 \\ \ell_{1},\ell_{2},\ell_{3}\in \mathbb{N}^{*}}}^{\infty} \xi_{\ell_{1},\ell_{2},\ell_{3}} t^{\ell_{1}\gamma _{1}}x^{\ell_{2}\gamma_{2}}y^{\ell_{3}\gamma_{3}}} &= \underbrace{ \xi_{000}}_{\ell _{1}+\ell_{2}+\ell_{3}=0}+\underbrace{\xi_{100} t^{\gamma_{1}}+\xi_{010} x^{\gamma_{2}}+\xi_{001} y^{\gamma_{3}}}_{\ell_{1}+\ell_{2}+\ell_{3}=1}+\cdots \\ &\quad+ \underbrace{ \sum_{i=0 }^{n} \sum_{j=0 }^{i} \xi_{n-i,i-j,j} t^{(n-i)\gamma_{1}}x^{(i-j)\gamma_{2}}y^{j\gamma_{3}}}_{\ell_{1}+\ell_{2}+\ell _{3}=n}+\cdots, \end{aligned} $$

where $\overline{\pmb{\gamma}}=(\gamma_{1}, \gamma_{2}, \gamma_{3})\in (0,1)^{3}$, $\{t, x, y \}$ is a set of nonnegative variables, and $\xi_{\ell_{1},\ell_{2},\ell_{3}}$ are real constants.

We point out here that the *γ̅*-Maclaurin can be naturally adapted to accommodate the problem under consideration by making the series coefficients as functions in extra variables as follows:

2.2 $$ \begin{aligned} {\sum_{\ell_{1}+\ell_{2}+\ell_{3}=0 }^{\infty} \xi_{\ell_{1},\ell_{2},\ell _{3}}(\overline{z}) t^{\ell_{1}\gamma_{1}}x^{\ell_{2}\gamma_{2}}y^{\ell_{3}\gamma_{3}}}. \end{aligned} $$

In what follows, we present some convergence theorems related to the *γ̅*-Maclaurin (2.1). A similar analysis can be adopted to expression (2.2). It should be mentioned here that comparable arguments were presented in a lower-dimensional fractional space in [52].

### Lemma 2.2

*If there exist*$t_{0}, x_{0}, y_{0}\in\mathbb{R}_{\geq0}$*such that the set*$\{\xi_{\ell_{1},\ell_{2},\ell_{3}} t_{0}^{\ell_{1}\gamma_{1}} x_{0}^{\ell_{2}\gamma_{2}} y_{0}^{\ell_{3}\gamma_{3}}: \ell_{1},\ell_{2},\ell _{3} \in \mathbb{N}^{*} \}$*is bounded*, *then theγ̅*-*Maclaurin* (2.1) *converges absolutely on*$\mathfrak {D}:=[0,t_{0})\times[0,x_{0})\times[0,y_{0})$.

### Proof

Let $t_{0}, x_{0}, y_{0} \in\mathbb{R}_{> 0}$. By assumption we have $|\xi_{\ell_{1},\ell_{2},\ell_{3}} t_{0}^{\ell_{1}\gamma_{1}} x_{0}^{\ell _{2}\gamma_{2}} y_{0}^{\ell_{3}\gamma_{3}}| \leq M$ for some $M\in\mathbb {R}_{>0}$ and all $\ell_{1},\ell_{2},\ell_{3}\in\mathbb{N}^{*}$. Now, for $(t,x,y)\in\mathfrak{D}-\{(0,0,0)\}$, set $0<\tau_{1}=t t_{0}^{-1}<1$, $0<\tau_{2}=x x_{0}^{-1}<1$, and $0<\tau_{3}=y y_{0}^{-1}<1$. Then

$$ \begin{aligned} {\sum_{\ell_{1}+\ell_{2}+\ell_{3}=0}^{\infty} \bigl\vert \xi_{\ell_{1},\ell_{2},\ell _{3}} t^{\ell_{1}\gamma_{1}}x^{\ell_{2}\gamma_{2}}y^{\ell_{3}\gamma_{3}} \bigr\vert }& = {\sum_{\ell_{1}+\ell_{2}+\ell_{3}=0}^{\infty} \bigl\vert \xi_{\ell_{1},\ell_{2},\ell_{3}} t_{0}^{\ell_{1}\gamma_{1}}x_{0}^{\ell_{2}\gamma _{2}}y_{0}^{\ell_{3}\gamma_{3}} \bigr\vert \tau_{1}^{\ell_{1}\gamma_{1}}\tau_{2}^{\ell_{2}\gamma_{2}} \tau_{3}^{\ell_{3}\gamma_{3}}} \\ &\leq{M \sum_{\ell_{1}+\ell_{2}+\ell_{3}=0}^{\infty} \tau_{1}^{\ell_{1}\gamma _{1}}\tau_{2}^{\ell_{2}\gamma_{2}} \tau_{3}^{\ell_{3}\gamma_{3}}} \\ &= {M \Biggl( \sum_{\ell_{1}=0}^{\infty} \tau_{1}^{\ell_{1}\gamma_{1}} \Biggr) \Biggl( \sum _{\ell_{2}=0}^{\infty}\tau_{2}^{\ell_{2}\gamma_{2}} \Biggr) \Biggl( \sum_{\ell _{3}=0}^{\infty} \tau_{3}^{\ell_{3}\gamma_{3}} \Biggr)} \\ &= {\frac{M}{(1-\tau_{1}^{\gamma_{1}})(1-\tau_{2}^{\gamma_{2}})(1-\tau _{3}^{\gamma_{3}})}< \infty}. \end{aligned} $$

Thus, the *γ̅*-Maclaurin (2.1) converges absolutely on $\mathfrak{D}$ as desired. Note that if one of $t_{0}$, $x_{0}$, or $y_{0}$ is zero, then a similar argument can be applied with fewer variables. □

### Theorem 2.3

*Theγ̅*-*Maclaurin* (2.1) *converges absolutely on either*$\mathbb{R}_{\geq0}^{3}$*or on*$\mathfrak {D}:=[0,r_{t})\times[0,r_{x})\times[0,r_{y})$*for some*$(r_{t}, r_{x}, r_{y}) \in\mathbb{R}^{3}_{\geq0}$. *In the latter case the set*$\mathcal {A}(t,x,y):=\{\xi_{\ell_{1},\ell_{2},\ell_{3}} t^{\ell_{1}\gamma_{1}}x^{\ell _{2}\gamma_{2}}y^{\ell_{3}\gamma_{3}}:\ell_{1},\ell_{2},\ell_{3}\in\mathbb{N}^{*}\}$*is unbounded outside*$\mathfrak{D}$.

### Proof

Let $\mathcal{B}:=\{(t,x,y)\in\mathbb{R}_{\geq 0}^{3}:\mathcal{A}(t,x,y)\text{ is bounded}\}$.

1. If $\mathcal{B}=\mathbb{R}_{\geq0}^{3}$, then the *γ̅*-Maclaurin converges absolutely on $\mathbb {R}_{\geq0}^{3}$ by the last lemma.
2. If $\mathcal{B}\neq\mathbb{R}_{\geq0}^{3}$, then $\partial\mathcal{B}$ is a nonempty set since $(0,0,0)\in\mathcal {B}$. Let $(r_{t}, r_{x}, r_{y})\in\partial\mathcal{B}$. By the definition of $(r_{t}, r_{x}, r_{y})$, if $(t,x,y)\in\mathfrak{D}$, then there exist $(t_{0},x_{0},y_{0})\in\mathcal{B}$ with $(t,x,y)\in [0,t_{0})\times[0,x_{0})\times[0,y_{0})$. Thus by the last lemma the *γ̅*-Maclaurin converges absolutely. On the other hand, if $(t,x,y)\in\mathbb{R}_{\geq0}^{3}$ with $(t,x,y)>(r_{t}, r_{x}, r_{y})$, then again by the definition there exist $(t_{1},x_{1},y_{1})\in\mathbb{R}_{\geq0}^{3}-\mathcal{B}$ with $r_{t}< t_{1}< t$, $r_{x}< x_{1}< x$, and $r_{y}< y_{1}< y$. Since $\mathcal {A}(t_{1},x_{1},y_{1})$ is unbounded, $\mathcal{A}(t,x,y)$ is unbounded as well.

 □

### Definition 2.4

The triple $(r_{t}, r_{x}, r_{y} ) \in\mathbb{R}_{\geq0}^{3}$ in the last theorem is called the triradius of convergence for *γ̅*-Maclaurin. Otherwise, we say that the triradius of convergence is infinity.

### Remark 1

By a suitable change of variable, it is easy to see that the series

$$\sum_{\substack{\ell_{1}+\ell_{2}+\ell_{3}=0 }}^{\infty} \xi_{\ell_{1},\ell _{2},\ell_{3}} t^{\ell_{1}}x^{\ell_{2}}y^{\ell_{3}} $$

with $(t,x,y)\in\mathbb {R}^{3}$ has a triradius of convergence $(r_{t}, r_{x}, r_{y})$ if and only if the *γ̅*-Maclaurin with $(t,x,y)\in\mathbb {R}_{\geq0}^{3}$ has a triradius of convergence $(r_{t}^{\gamma _{1}^{-1}}, r_{x}^{\gamma_{2}^{-1}}, r_{y}^{\gamma_{3}^{-1}} )$.

### Remark 2

It is worth mentioning that the *γ̅*-Maclaurin is the Cauchy product of fractional power series, after rearrangement, in the domain of absolute convergence. Nevertheless, the *γ̅*-Maclaurin enables us to add finite elements on each term instead of adding an entire row or column with infinite elements.

$$ {\sum_{\substack{\ell_{1}+\ell_{2}+\ell_{3}=0 }}^{\infty} \xi_{\ell_{1},\ell _{2},\ell_{3}} t^{\ell_{1}\gamma_{1}}x^{\ell_{2}\gamma_{2}}y^{\ell_{3}\gamma_{3}}}= \Biggl( \sum_{\ell_{1}=0}^{\infty}a_{\ell_{1}} t^{\ell_{1}\gamma_{1}} \Biggr) \Biggl( \sum_{\ell_{2}=0}^{\infty}b_{\ell_{2}} x^{\ell_{2}\gamma_{2}} \Biggr) \Biggl( \sum_{\ell_{3}=0}^{\infty}c_{\ell_{3}} y^{\ell_{3}\gamma_{3}} \Biggr), $$

where $\xi_{\ell_{1},\ell_{2},\ell_{3}}=a_{\ell_{1}}b_{\ell_{2}}c_{\ell_{3}}$. These three fractional power series in a single variable will be called the components of *γ̅*-Maclaurin. Moreover, the *γ̅*-Maclaurin can be rewritten as the triple sum

$$ \sum_{\ell_{1}=0}^{\infty}\sum _{\ell_{2}=0}^{\ell_{1}}\sum _{\ell_{3}=0}^{\ell _{2}} \xi_{\ell_{1}-\ell_{2},\ell_{2}-\ell_{3},\ell_{3}} t^{(\ell_{1}-\ell_{2})\gamma _{1}}x^{(\ell_{2}-\ell_{3})\gamma_{2}}y^{\ell_{3}\gamma_{3}}. $$

### Theorem 2.5

*If the components ofγ̅*-*Maclaurin converge absolutely at*$t_{0}, x_{0}, y_{0}>0$, *respectively*, *then theγ̅*-*Maclaurin*$\sum_{\ell_{1}=0}^{\infty}\sum_{\ell_{2}=0}^{\ell _{1}}\sum_{\ell_{3}=0}^{\ell_{2}} a_{\ell_{1}-\ell_{2}}b_{\ell_{2}-\ell_{3}}c_{\ell _{3}} t^{(\ell_{1}-\ell_{2})\gamma_{1}}x^{(\ell_{2}-\ell_{3})\gamma_{2}}y^{\ell _{3}\gamma_{3}}$*converges absolutely on*$\mathfrak{D}=[0,t_{0})\times [0,x_{0})\times[0,y_{0})$.

### Proof

By assumption, $\sum_{\ell_{1}=0}^{\infty} \vert a_{\ell_{1}}t^{\ell _{1}\gamma _{1}} \vert < L_{1}$, $\sum_{\ell_{2}=0}^{\infty} \vert b_{\ell_{2}}x^{\ell _{2}\gamma_{2}} \vert < L_{2}$, and $\sum_{\ell_{3}=0}^{\infty} \vert c_{\ell _{3}}y^{\ell_{3}\gamma_{3}} \vert < L_{3}$ for some $L_{1}, L_{2}, L_{3} \in\mathbb {R}_{>0}$. For each $\ell_{1} \in\mathbb{N}$, let $E_{\ell _{1}}(t_{0},x_{0},y_{0})=\sum_{\ell_{2}=0}^{\ell_{1}}\sum_{\ell_{3}=0}^{\ell_{2}} a_{\ell_{1}-\ell_{2}}b_{\ell_{2}-\ell_{3}}c_{\ell_{3}} t_{0}^{(\ell_{1}-\ell _{2})\gamma _{1}}x_{0}^{(\ell_{2}-\ell_{3})\gamma_{2}}y_{0}^{\ell_{3}\gamma_{3}}$. Then

$$\begin{aligned} \bigl\vert E_{\ell_{1}}(t_{0},x_{0},y_{0}) \bigr\vert &= { \Biggl\vert \sum_{\ell _{2}=0}^{\ell_{1}} \sum_{\ell_{3}=0}^{\ell_{2}} a_{\ell_{1}-\ell_{2}}b_{\ell_{2}-\ell _{3}}c_{\ell_{3}} t_{0}^{(\ell_{1}-\ell_{2})\gamma_{1}}x_{0}^{(\ell_{2}-\ell_{3})\gamma _{2}}y_{0}^{\ell_{3}\gamma_{3}} \Biggr\vert } \\ &\leq{\sum_{\ell_{2}=0}^{\ell_{1}}\sum _{\ell_{3}=0}^{\ell_{2}} \bigl\vert a_{\ell _{1}-\ell_{2}}t_{0}^{(\ell_{1}-\ell_{2})\gamma_{1}} \bigr\vert \bigl\vert b_{\ell_{2}-\ell_{3}} x_{0}^{(\ell_{2}-\ell_{3})\gamma_{2}} \bigr\vert \bigl\vert c_{\ell_{3}}y_{0}^{\ell_{3}\gamma _{3}} \bigr\vert } \\ &\leq{\sum_{\ell_{2}=0}^{\ell_{1}} \bigl\vert a_{\ell_{1}-\ell_{2}}t_{0}^{(\ell _{1}-\ell_{2})\gamma_{1}} \bigr\vert \sum _{\ell_{3}=0}^{\ell_{2}} \bigl\vert b_{\ell_{2}-\ell_{3}} x_{0}^{(\ell_{2}-\ell_{3})\gamma_{2}} \bigr\vert \bigl\vert c_{\ell_{3}}y_{0}^{\ell_{3}\gamma _{3}} \bigr\vert } \\ &\leq{\sum_{\ell_{2}=0}^{\ell_{1}} \bigl\vert a_{\ell_{1}-\ell_{2}}t_{0}^{(\ell _{1}-\ell_{2})\gamma_{1}} \bigr\vert \Biggl(\sum _{\ell_{3}=0}^{\ell_{2}} \bigl\vert b_{\ell _{2}-\ell_{3}} x_{0}^{(\ell_{2}-\ell_{3})\gamma_{2}} \bigr\vert \Biggr) \Biggl(\sum_{\ell_{3}=0}^{\ell_{2}} \bigl\vert c_{\ell_{3}}y_{0}^{\ell_{3}\gamma_{3}} \bigr\vert \Biggr)} \\ &\leq{\sum_{\ell_{2}=0}^{\infty} \bigl\vert a_{\ell_{1}-\ell_{2}}t_{0}^{(\ell_{1}-\ell _{2})\gamma_{1}} \bigr\vert \Biggl(\sum _{\ell_{3}=0}^{\infty} \bigl\vert b_{\ell_{2}-\ell_{3}} x_{0}^{(\ell_{2}-\ell_{3})\gamma_{2}} \bigr\vert \Biggr) \Biggl(\sum_{\ell_{3}=0}^{\infty} \bigl\vert c_{\ell_{3}}y_{0}^{\ell_{3}\gamma_{3}} \bigr\vert \Biggr)} \\ &< L_{1}L_{2}L_{3} < \infty. \end{aligned}$$

This shows that the sequence $\{E_{\ell_{1}}(t_{0},x_{0},y_{0})\}$ is bounded. Therefore the *γ̅*-Maclaurin converges absolutely on $\mathfrak{D}$ by Lemma 2.2, as desired. □

### Remark 3

It is evident that if the above fractional power series components of the *γ̅*-Maclaurin have a radius of convergence $r_{t}$, $r_{x}$, $r_{y}$, respectively, then the *γ̅*-Maclaurin has a triradius of convergence $(r_{t}, r_{x},r_{y})$.

### Notation 1

For simplicity, we will alternatively write $\varGamma (\ell\gamma+1 )$ as $\varGamma_{\gamma}(\ell)$.

Now, as our goal is to furnish an analytical solution of higher-dimensional FPDEs, we take into account the Caputo fractional derivative, which is defined for an appropriate function as follows:

2.3 $$ \mathcal{D}^{\gamma}_{t} \bigl[ \omega(t,x,y) \bigr]= {\frac{1}{\varGamma (1-\alpha)} \int_{0}^{t} \frac{\partial\omega(\tau,x,y)}{\partial\tau} \frac{d\tau}{ (t-\tau)^{\gamma}}}, $$

where $\gamma\in(0,1)$ is the Caputo fractional derivative order. Accordingly, immediate computations lead to

2.4 $$ \mathcal{D}^{\gamma}_{t} \bigl[t^{c} \bigr]=\left \{ \textstyle\begin{array}{l@{\quad}l} {\frac{\varGamma(c+1 )}{\varGamma(c-\gamma+1 )} t^{c-\gamma}}, & c>0,\\ 0, & c=0. \end{array}\displaystyle \right . $$

The following theorem shows the mixed Caputo fractional derivatives of analytic functions in fractional sense of *γ̅*-Maclaurin [53, 54].

### Theorem 2.6

*Let*$\omega(t,x,y)$*have aγ̅*-*Maclaurin on*$\mathfrak{D}=[0,r_{t})\times[0,r_{y}) \times[0,r_{t})$. *If*$\mathcal {D}^{i\gamma_{1}}_{t}\mathcal{D}^{j\gamma_{2}}_{x}\mathcal{D}^{k\gamma _{3}}_{y} [\omega(t,x,y) ]\in\mathcal{C} ((0,r_{t})\times(0,r_{x}) \times(0,r_{y}) )$*for*$i, j, k\in\mathbb{N}$. *Then*

2.5 $$ \begin{gathered}[b]\mathcal{D}^{i\gamma_{1}}_{t} \mathcal{D}^{j\gamma_{2}}_{x}\mathcal{D}^{k\gamma_{3}}_{y} \bigl[\omega(t,x,y) \bigr] \\\quad= \sum_{\ell_{1}+\ell_{2}+\ell _{3}=0 }^{\infty} \xi_{i+\ell_{1},j+\ell_{2},k+\ell_{3}} \frac{\varGamma_{\gamma _{1}} (i+\ell_{1} )\varGamma_{\gamma_{2}} (j+\ell_{2} )\varGamma _{\gamma_{3}} (k+\ell_{3} )}{\varGamma_{\gamma_{1}} (\ell_{1} )\varGamma_{\gamma_{2}} (\ell_{2} )\varGamma_{\gamma_{3}} (\ell _{3} )} t^{\ell_{1}\gamma_{1} }x^{\ell_{2}\gamma_{2}}y^{\ell_{3}\gamma_{3}}. \end{gathered} $$

In (2.5), upon substituting $(t,x,y)=(0,0,0)$, we obtain the coefficients in terms of the mixed Caputo fractional derivatives as

2.6 $$ \xi_{\ell_{1},\ell_{2},\ell_{3}}= \frac{\mathcal{D}^{\ell _{1}\gamma _{1}}_{t}\mathcal{D}^{\ell_{2}\gamma_{2}}_{x}\mathcal{D}^{\ell_{3}\gamma _{3}}_{y} [\omega(0,0,0) ]}{\varGamma_{\gamma_{1}} (\ell_{1} )\varGamma_{\gamma_{2}} (\ell_{2} ) \varGamma_{\gamma_{3}} (\ell_{3} )}, $$

which is the fractional version of the classical multivariate Maclaurin coefficients.

## An application of *γ̅*-Maclaurin series

In this section, the *γ̅*-Maclaurin series is merged in the power series technique to furnish analytically the solution of the PDEs endowed with multimemory indices. It is worth recalling here some necessary fractional functions that will be frequently used in the sequel:

- Mittag-Leffler function: ${ E_{\gamma}(t)= \sum_{\ell =0}^{\infty}\frac{t^{\ell}}{\varGamma_{\gamma}(\ell)}}$,
- Trigonometric functions: ${ \sin_{\gamma}(t)= \sum_{\ell =0}^{\infty}\frac{(-1)^{\ell} t^{2\ell+1}}{\varGamma_{\gamma}(2\ell +1)}}$, ${ \cos_{\gamma}(t)= \sum_{\ell=0}^{\infty}\frac{(-1)^{\ell} t^{2\ell}}{\varGamma_{\gamma}(2\ell)} }$,
- Hyperbolic functions: ${ \sinh_{\gamma}(t)= \sum_{\ell =0}^{\infty}\frac{ t^{2\ell+1}}{\varGamma_{\gamma}(2\ell+1)} }$, ${ \cosh_{\gamma}(t)= \sum_{\ell=0}^{\infty}\frac{ t^{2\ell}}{\varGamma _{\gamma}(2\ell)}}$.

### Example 1

In our first illustrative example, we consider the following hyperbolic *γ̅*-wave-like equation:

3.1 $$ \mathcal{D}_{t}^{2\gamma_{1}}\bigl[ \omega(t,x,y)\bigr]=\frac{1}{2} \bigl(\mathcal{D}_{x}^{2\gamma_{2}} \bigl[\omega(t,x,y)\bigr] + \mathcal{D}_{y}^{2\gamma_{3}}\bigl[ \omega(t,x,y)\bigr] \bigr), $$

constrained by the fractional initial conditions

3.2 $$ \begin{gathered} \omega(0,x,y) = \sin_{\gamma_{2}}\bigl(x^{\gamma_{2}}\bigr) \cos_{\gamma _{3}} \bigl(y^{\gamma_{3}}\bigr) + \cos_{\gamma_{2}}\bigl(x^{\gamma_{2}} \bigr) \sin_{\gamma _{3}}\bigl(y^{\gamma_{3}}\bigr), \\ \mathcal{D}_{t}^{\gamma_{1}}\bigl[\omega(0,x,y)\bigr] = \sin_{\gamma_{2}}\bigl(x^{\gamma _{2}}\bigr) \sin_{\gamma_{3}} \bigl(y^{\gamma_{3}}\bigr) - \cos_{\gamma_{2}}\bigl(x^{\gamma_{2}} \bigr) \cos_{\gamma_{3}}\bigl(y^{\gamma_{3}}\bigr). \end{gathered} $$

We presume that the solution exists analytically in the form (2.1). Now we substitute the proper formulas from Theorem 2.6 into equations (3.1)–(3.2) and compare the coefficients of identical monomials in both parties to get the following recurrence relation

3.3 $$ {\frac{\varGamma_{\gamma_{1}}(\ell_{1}+2)}{\varGamma _{\gamma_{1}}(\ell_{1})} \xi_{\ell_{1}+2,\ell_{2},\ell_{3}}} = \frac{1}{2} \biggl( {\frac{\varGamma _{\gamma_{2}}(\ell_{2}+2)}{\varGamma_{\gamma_{2}}(\ell_{2})} \xi_{\ell_{1},\ell _{2}+2,\ell_{3}}}+ { \frac{\varGamma_{\gamma_{3}}(\ell_{3}+2)}{\varGamma_{\gamma _{3}}(\ell_{3})} \xi_{\ell_{1},\ell_{2},\ell_{3}+2}} \biggr) $$

with initial coefficients

3.4 $$ \begin{gathered} \xi_{0,2\ell_{2}+1,2\ell_{3}} = \frac{(-1)^{\ell_{2}+\ell_{3}}}{\varGamma _{\gamma_{2}}(2\ell_{2}+1) \varGamma_{\gamma_{3}}(2\ell_{3})},\\ \xi_{0,2\ell _{2},2\ell_{3}+1} = \frac{(-1)^{\ell_{2}+\ell_{3}}}{\varGamma_{\gamma_{2}}(2\ell _{2}) \varGamma_{\gamma_{3}}(2\ell_{3}+1)}, \\ \xi_{1,2\ell_{2},2\ell_{3}} = \frac{(-1)^{\ell_{2}+\ell_{3}+1}}{\varGamma _{\gamma_{1}}(1) \varGamma_{\gamma_{2}}(2\ell_{2}) \varGamma_{\gamma_{3}}(2\ell _{3})},\\ \xi_{1,2\ell_{2}+1,2\ell_{3}+1}= \frac{(-1)^{\ell_{2}+\ell_{3}}}{\varGamma _{\gamma_{1}}(1) \varGamma_{\gamma_{2}}(2\ell_{2}+1) \varGamma_{\gamma _{3}}(2\ell_{3}+1)}. \end{gathered} $$

Next, in light of the initial coefficients (3.4), we recursively solve (3.3) to obtain the following general form of the series coefficients:

3.5 $$\begin{aligned}& \xi_{2\ell_{1},2\ell_{2}+1,2\ell_{3}}= \frac{(-1)^{\ell_{1}+\ell_{2}+\ell _{3}}}{\varGamma_{\gamma_{1}}(2\ell_{1}) \varGamma_{\gamma_{2}}(2\ell_{2}+1) \varGamma_{\gamma_{3}}(2\ell_{3})}, \\& \xi_{2\ell_{1},2\ell_{2},2\ell_{3}+1}= \frac{(-1)^{\ell_{1}+\ell_{2}+\ell _{3}}}{\varGamma_{\gamma_{1}}(2\ell_{1}) \varGamma_{\gamma_{2}}(2\ell_{2}) \varGamma_{\gamma_{3}}(2\ell_{3}+1)}, \\& \xi_{2\ell_{1}+1,2\ell_{2},2\ell_{3}}= -\frac{(-1)^{\ell_{1}+\ell_{2}+\ell _{3}}}{\varGamma_{\gamma_{1}}(2\ell_{1}+1) \varGamma_{\gamma_{2}}(2\ell_{2}) \varGamma_{\gamma_{3}}(2\ell_{3})}, \\& \xi_{2\ell_{1}+1,2\ell_{2}+1,2\ell_{3}+1} = \frac{(-1)^{\ell_{1}+\ell_{2}+\ell _{3}}}{\varGamma_{\gamma_{1}}(2\ell_{1}+1) \varGamma_{\gamma_{2}}(2\ell_{2}+1) \varGamma_{\gamma_{3}}(2\ell_{3}+1)}, \\& \xi_{\ell_{1},\ell_{2},\ell_{3}}= 0 \quad\text{otherwise}. \end{aligned}$$

Finally, we substitute the resulted coefficient (3.5) into the *γ̅*-Maclaurin series to get

3.6 $$ \begin{aligned}[b] \omega(t,x,y)& = {\sum _{\substack{\ell_{1}+\ell_{2}+\ell_{3}=0}}^{\infty} \biggl[\frac{(-1)^{\ell _{1}+\ell_{2}+\ell_{3}} t^{2\ell_{1}\gamma_{1}}x^{(2\ell _{2}+1)\gamma_{2}}y^{2\ell_{3}\gamma_{3}}}{\varGamma_{\gamma_{1}}(2\ell_{1}) \varGamma_{\gamma_{2}}(2\ell_{2}+1) \varGamma_{\gamma_{3}}(2\ell_{3})} } \\ &\quad+ { \frac{(-1)^{\ell_{1}+\ell_{2}+\ell_{3}} t^{2\ell_{1}\gamma_{1}}x^{2\ell _{2}\gamma_{2}}y^{(2\ell_{3}+1)\gamma_{3}}}{\varGamma_{\gamma_{1}}(2\ell_{1}) \varGamma_{\gamma_{2}}(2\ell_{2}) \varGamma_{\gamma_{3}}(2\ell_{3}+1)}} \\ &\quad- {\frac{(-1)^{\ell_{1}+\ell_{2}+\ell_{3}} t^{(2\ell_{1}+1)\gamma _{1}}x^{2\ell_{2}\gamma_{2}}y^{2\ell_{3}\gamma_{3}}}{\varGamma_{\gamma_{1}}(2\ell _{1}+1) \varGamma_{\gamma_{2}}(2\ell_{2}) \varGamma_{\gamma_{3}}(2\ell_{3})}}\\ &\quad+ {\frac{(-1)^{\ell_{1}+\ell_{2}+\ell_{3}} t^{(2\ell_{1}+1)\gamma _{1}}x^{(2\ell _{2}+1)\gamma_{2}}y^{(2\ell_{3}+1)\gamma_{3}}}{\varGamma_{\gamma_{1}}(2\ell_{1}+1) \varGamma_{\gamma_{2}}(2\ell_{2}+1) \varGamma_{\gamma_{3}}(2\ell_{3}+1)}} \biggr]. \end{aligned} $$

As each of these sums converges absolutely in $[0,\infty)^{3}$ by the ratio test, Remark 2 implies that each sum can be written as a Cauchy product of three series. For example, for the first sum, we have

$$ \begin{gathered} {\sum_{\substack{\ell_{1}+\ell_{2}+\ell_{3}=0}}^{\infty} \frac{(-1)^{\ell _{1}+\ell_{2}+\ell_{3}} t^{2\ell_{1}\gamma_{1}}x^{(2\ell_{2}+1)\gamma_{2}}y^{2\ell _{3}\gamma_{3}}}{\varGamma_{\gamma_{1}}(2\ell_{1}) \varGamma_{\gamma_{2}}(2\ell _{2}+1) \varGamma_{\gamma_{3}}(2\ell_{3})}} \\\quad= \sum_{\ell_{1}=0}^{\infty} \frac {(-1)^{\ell_{1}} t^{2\ell_{1}\gamma_{1}}}{\varGamma_{\gamma_{1}}(2\ell_{1})} \sum _{\ell_{2}=0}^{\infty} \frac{(-1)^{\ell_{2}} x^{(2\ell_{2}+1)\gamma _{2}}}{\varGamma_{\gamma_{2}}(2\ell_{2}+1)} \sum_{\ell_{3}=0}^{\infty} \frac{(-1)^{\ell_{3}} y^{2\ell_{3}\gamma _{3}}}{\varGamma_{\gamma_{3}}(2\ell_{3})} \\ \quad= \cos_{\gamma_{1}}\bigl(t^{\gamma_{1}}\bigr)\sin_{\gamma_{2}} \bigl(x^{\gamma_{2}}\bigr)\cos_{\gamma_{3}}\bigl(y^{\gamma_{3}} \bigr). \end{gathered} $$

Therefore the solution (3.6) reduced to the following closed form:

3.7 $$ \begin{aligned}[b] \omega(t,x,y)&= { \cos_{\gamma_{1}}\bigl(t^{\gamma_{1}}\bigr)\sin_{\gamma_{2}} \bigl(x^{\gamma _{2}}\bigr)\cos_{\gamma_{3}}\bigl(y^{\gamma_{3}} \bigr)+\cos_{\gamma_{1}}\bigl(t^{\gamma_{1}}\bigr)\cos_{\gamma_{2}} \bigl(x^{\gamma_{2}}\bigr)\sin_{\gamma_{3}}\bigl(y^{\gamma_{3}} \bigr)} \\ &\quad- {\sin_{\gamma _{1}}\bigl(t^{\gamma_{1}}\bigr)\cos_{\gamma_{2}} \bigl(x^{\gamma_{2}}\bigr)\cos_{\gamma_{3}}\bigl(y^{\gamma _{3}} \bigr)}+ {\sin_{\gamma_{1}}\bigl(t^{\gamma_{1}}\bigr) \sin_{\gamma_{2}}\bigl(x^{\gamma_{2}}\bigr)\sin_{\gamma_{3}} \bigl(y^{\gamma_{3}}\bigr)}. \end{aligned} $$

As a particular case, if $\overline{\pmb{\gamma}}\rightarrow\overline {1}$, then we obtain the solution of the wave-like equation in the integer case:

3.8 $$ \omega(t,x,y)=\sin(x+y-t). $$

**Figure 1:**
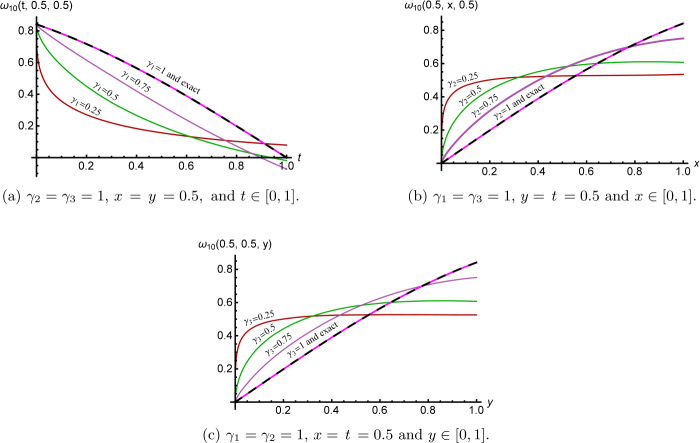
Cross-sections of the 10th approximate solution (3.6)

Figure 1 clarifies the cross-sections of the 10th approximate *γ̅*-Maclaurin solution (3.6) for several values of $\overline{\pmb{\gamma}} \in(0,1)^{3}$. Their performance shows that the *γ̅*-Maclaurin solution depends continuously on the fractional derivative parameters to attain the integer case solution, which in turn reflects some information about memory.

### Example 2

Next, we consider the following *γ̅*-heat equation:

3.9 $$ \mathcal{D}^{\gamma_{1}}_{t} \bigl[ \omega(t,x,y) \bigr]=\frac{1}{2} \bigl(y^{2\gamma_{3}} \mathcal{D}^{2\gamma_{2}}_{x} \bigl[\omega(t,x,y) \bigr]+x^{2\gamma_{2}}\mathcal{D}^{2\gamma_{3}}_{y} \bigl[ \omega(t,x,y) \bigr] \bigr), $$

constrained by the fractional initial condition

3.10 $$ \omega(0,x,y)=y^{2\gamma_{3}}. $$

Again, we presume that the solution exists analytically in the form (2.1). Now we substitute the proper formulas from Theorem 2.6 into equations (3.9)–(3.10) and compare the coefficients of identical monomials in both parties to get the following recurrence relations for each $\ell_{i}\geq0$

3.11 $$ \begin{gathered}[b]\frac{\varGamma_{\gamma_{1}}(\ell_{1}+1)}{\varGamma _{\gamma_{1}}(\ell_{1})} \xi_{\ell_{1}+1,\ell_{2},\ell_{3}}\\\quad= \textstyle\begin{cases} {0} ,& {\ell_{2}, \ell_{3}< 2 }, \\ {\frac{\varGamma_{\gamma_{2}}(\ell_{2}+2)}{2\varGamma_{\gamma_{2}}(\ell_{2})} \xi_{\ell_{1},\ell_{2}+2,\ell_{3}-2}}, & {0 \leq\ell_{2}< 2, \ell_{3}\geq2 },\\ {\frac{\varGamma_{\gamma_{3}}(\ell_{3}+2)}{2\varGamma_{\gamma_{3}}(\ell_{3})} \xi_{\ell_{1},\ell_{2}-2,\ell_{3}+2}}, & {\ell_{2}\geq2, 0\leq\ell_{3}< 2 },\\ {\frac{\varGamma_{\gamma_{2}}(\ell_{2}+2)}{2\varGamma_{\gamma_{2}}(\ell_{2})} \xi_{\ell_{1},\ell_{2}+2,\ell_{3}-2}+\frac{\varGamma_{\gamma_{3}}(\ell _{3}+2)}{2\varGamma_{\gamma_{3}}(\ell_{3})} \xi_{\ell_{1},\ell_{2}-2,\ell_{3}+2}}, & {\ell_{2}, \ell_{3}\geq2 }, \end{cases}\displaystyle \end{gathered} $$

with initial coefficient $\xi_{0,0,2}=1$. Next, we recursively solve (3.11) to obtain the following general form of the series coefficients:

3.12 $$ \begin{gathered} \xi_{2\ell_{1},0,2}= \frac{\varGamma^{\ell_{1}}_{\gamma_{2}}(2) \varGamma ^{\ell_{1}}_{\gamma_{3}}(2)}{2^{2\ell_{1}}\varGamma_{\gamma_{1}}(2\ell_{1})}, \\ \xi_{2\ell_{1}+1,2,0} =\frac{\varGamma^{\ell_{1}}_{\gamma_{2}}(2) \varGamma ^{\ell_{1}+1}_{\gamma_{3}}(2)}{2^{2\ell_{1}+1}\varGamma_{\gamma_{1}}(2\ell _{1}+1)}, \\ \xi_{\ell_{1},\ell_{2},\ell_{3}}=0\quad\text{otherwise}. \end{gathered} $$

We now substitute the resulting coefficient (3.12) into the *γ̅*-Maclaurin series to get

3.13 $$ \begin{aligned}[b] \omega(t,x,y)& = {\sum _{\substack{\ell_{1}=0}}^{\infty} \xi_{2\ell_{1},0,2} t^{2\ell_{1}\gamma_{1}}y^{2\gamma_{3}}+\sum_{\substack{\ell_{1}=0 }}^{\infty} \xi_{2\ell_{1}+1,2,0} t^{(2\ell_{1}+1)\gamma_{1}}x^{2\gamma_{2}}} \\ & = {y^{2\gamma_{3}}\sum_{\ell_{1}=0}^{\infty} \frac{\varGamma^{\ell _{1}}_{\gamma_{2}}(2) \varGamma^{\ell_{1}}_{\gamma_{3}}(2)}{2^{2\ell_{1}}\varGamma _{\gamma_{1}}(2\ell_{1})} t^{2\ell_{1}\gamma_{1}}} + {x^{2\gamma_{2}}\sum _{\ell_{1}=0}^{\infty}\frac{\varGamma^{\ell _{1}}_{\gamma_{2}}(2) \varGamma^{\ell_{1}+1}_{\gamma_{3}}(2)}{2^{2\ell _{1}+1}\varGamma_{\gamma_{1}}(2\ell_{1}+1)} t^{(2\ell_{1}+1)\gamma_{1}}} \\ & = y^{2\gamma_{3}}\cosh_{\gamma_{1}} \biggl( \frac{\varGamma_{\gamma _{2}}^{0.5}(2)\varGamma^{0.5}_{\gamma}(2)}{2} t^{\gamma_{1}} \biggr)\\&\quad+ \biggl(\frac{\varGamma_{\gamma _{3}}(2)}{\varGamma_{\gamma_{2}}(2)} \biggr)^{0.5} x^{2\gamma_{2}} \sinh_{\gamma_{1}} \biggl( \frac{\varGamma_{\gamma _{2}}^{0.5}(2)\varGamma^{0.5}_{\gamma_{3}}(2)}{2} t^{\gamma_{1}} \biggr). \end{aligned} $$

As a particular case, if $\overline{\pmb{\gamma}}\rightarrow\overline {1}$, then we obtain the solution of the heat equation in the integer case:

3.14 $$ \begin{aligned} \omega(t,x,y)&=y^{2} \cosh(t)+x^{2}\sinh(t). \end{aligned} $$

**Figure 2:**
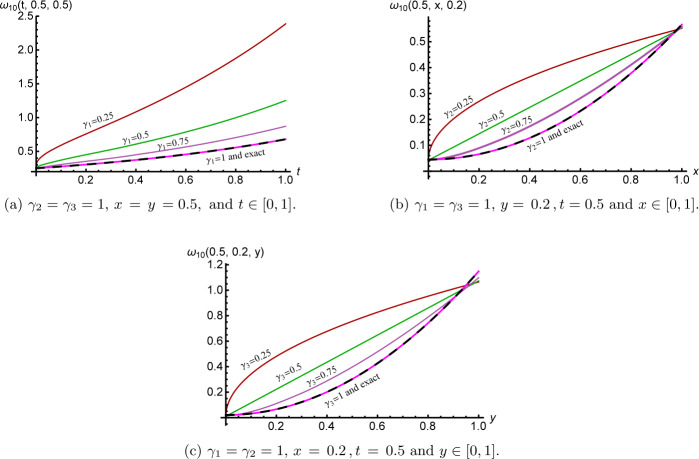
Cross-sections of the 10th approximate solution (3.13)

Figure 2 clarifies the cross-sections of the 10th approximate *γ̅*-Maclaurin solution (3.13) for several values of $\overline{\pmb{\gamma}} \in(0,1)^{3}$. Again, their performance shows that the *γ̅*-Maclaurin solution depends continuously on the fractional derivative parameters to attain the integer case solution, which in turn reflects some information about memory.

### Example 3

Finally, we consider the following *γ̅*-wave-like equation in 4D:

3.15 $$ \begin{gathered}[b] \mathcal{D}^{2\gamma_{1}}_{t} \bigl[\omega(t,x,y,z) \bigr]\\\quad= x^{2\gamma _{2}}+y^{2\gamma_{3}}+z^{2}\\\quad\quad+ \frac{1}{2} \bigl(x^{2\gamma_{2}}\mathcal{D}^{2\gamma _{2}}_{x}1 \bigl[\omega(t,x,y,z) \bigr]+y^{2\gamma_{3}}\mathcal{D}^{2\gamma _{3}}_{y} \bigl[\omega(t,x,y,z) \bigr]+z^{2} \omega_{zz}(t,x,y,z) \bigr), \end{gathered} $$

constrained by the fractional initial conditions

3.16 $$ \begin{gathered} \omega(0,x,y,z) = 0, \\ \mathcal{D}_{t}^{\gamma_{1}}\bigl[\omega(0,x,y,z)\bigr] = x^{2\gamma_{2}}+y^{2\gamma_{3}}-z^{2}. \end{gathered} $$

We presume that the solution exists analytically in the form (2.2). We substitute the proper formulas from Theorem 2.6 into equations (3.15)–(3.16) and compare the coefficients of identical monomials in both parties to get the following recurrence-differential equations for each $\ell_{1}\geq0$:

3.17 $$ \begin{gathered}[b] {\frac{\varGamma_{\gamma_{1}}(\ell_{1}+2)}{\varGamma _{\gamma_{1}}(\ell_{1})} \xi_{\ell_{1}+2,\ell_{2},\ell_{3}}(z)}\\\quad= \textstyle\begin{cases} {\frac{1}{2}z^{2}\xi^{\prime\prime}_{\ell_{1},\ell_{2},\ell_{3}}(z)+ z^{2}}, & {\ell_{2},\ell_{3} < 2 }, \\ \frac{1}{2} ( \frac{\varGamma_{\gamma_{2}}(\ell_{2})}{\varGamma _{\gamma_{2}}(\ell_{2}-2)} \xi_{\ell_{1},\ell_{2},\ell_{3}}(z)\\ \quad+ \frac{\varGamma_{\gamma_{3}}(\ell_{3})}{\varGamma_{\gamma_{3}}(\ell_{3}-2)} \xi _{\ell_{1},\ell_{2},\ell_{3}}(z)+z^{2}\xi^{\prime\prime}_{\ell_{1},\ell_{2},\ell _{3}}(z) )+2, & {\ell_{2},\ell_{3} = 2 },\\ \frac{1}{2} (\frac{\varGamma_{\gamma_{2}}(\ell_{2})}{\varGamma_{\gamma _{2}}(\ell_{2}-2)} \xi_{\ell_{1},\ell_{2},\ell_{3}}(z)\\ \quad+ \frac{\varGamma_{\gamma_{3}}(\ell_{3})}{\varGamma_{\gamma_{3}}(\ell_{3}-2)} \xi _{\ell_{1},\ell_{2},\ell_{3}}(z)+z^{2}\xi^{\prime\prime}_{\ell_{1},\ell_{2},\ell _{3}}(z) )+1 ,& {\substack{\ell_{2}=2\ \&\ \ell_{3}>2, \\ \ell_{2}>2\ \&\ \ell _{3}=2,}}\\ {\frac{1}{2}} ( {\frac{\varGamma_{\gamma_{2}}(\ell_{2})}{\varGamma _{\gamma_{2}}(\ell_{2}-2)} \xi_{\ell_{1},\ell_{2},\ell_{3}}(z)}\\\quad+ {\frac{\varGamma_{\gamma_{3}}(\ell_{3})}{\varGamma_{\gamma_{3}}(\ell_{3}-2)} \xi_{\ell_{1},\ell_{2},\ell_{3}}(z)+z^{2}\xi^{\prime\prime}_{\ell_{1},\ell_{2},\ell _{3}}(z)} ), & \text{otherwise}, \end{cases}\displaystyle \end{gathered} $$

with initial coefficients

3.18 $$ \begin{gathered} \xi_{0,\ell_{2},\ell_{3}}(z)=0, \qquad\xi_{1,0,0}(z)=-\frac{1}{\varGamma_{\gamma _{1}}(1)}z^{2}, \\ \xi_{1,2,0}(z)= \xi_{1,0,2}=\frac{1}{\varGamma_{\gamma_{1}}(1)},\qquad\xi _{1,\ell_{2},\ell_{3}}(z)=0\quad\text{otherwise}. \end{gathered} $$

In light of the initial coefficients (3.18), we recursively solve (3.17) to obtain the following general form of the series coefficients

3.19 $$ \begin{gathered} \xi_{\ell_{1},0,0}(z)= \frac{(-1)^{\ell_{1}} }{\varGamma_{\gamma_{1}}(\ell _{1})} z^{2}, \\ \xi_{2\ell_{1},2,0}(z)= {\frac{\varGamma^{\ell_{1}-1}_{\gamma _{2}}(2)}{2^{\ell_{1}-1} \varGamma_{\gamma_{1}}(2\ell_{1})}},\qquad\xi_{2\ell _{1}+1,2,0}(z)= { \frac{\varGamma^{\ell_{1}}_{\gamma_{2}}(2)}{2^{\ell_{1}} \varGamma_{\gamma_{1}}(2\ell_{1}+1)}}, \\ \xi_{2\ell_{1},0,2}(z)= {\frac{\varGamma^{\ell_{1}-1}_{\gamma _{3}}(2)}{2^{\ell_{1}-1} \varGamma_{\gamma_{1}}(2\ell_{1})}},\qquad\xi_{2\ell _{1}+1,0,2}(z)= { \frac{\varGamma^{\ell_{1}}_{\gamma_{3}}(2)}{2^{\ell_{1}} \varGamma_{\gamma_{1}}(2\ell_{1}+1)}}, \\ \xi_{\ell_{1},\ell_{2},\ell_{3}}(z)=0\quad\text{otherwise}. \end{gathered} $$

We now substitute the resulting coefficients (3.19) into the *γ̅*-Maclaurin series to get

3.20 $$ \begin{aligned}[b] \omega(t,x,y,z) &= {\sum _{\substack{\ell_{1}=1}}^{\infty} \xi_{\ell _{1},0,0}(z) t^{\ell_{1}\gamma_{1}}+\sum_{\substack{\ell_{1}=0 }}^{\infty} \xi _{\ell_{1},2,0}(z) t^{\ell_{1}\gamma_{1}}x^{2\gamma_{2}}+\sum _{\substack{\ell _{1}=0 }}^{\infty} \xi_{\ell_{1},0,2}(z) t^{\ell_{1}\gamma_{1}}y^{2\gamma_{3}}} \\ &=\sum_{\substack{\ell_{1}=1}}^{\infty} \frac{(-1)^{\ell_{1}}}{\varGamma _{\gamma_{1}}(\ell_{1})} z^{2}t^{\ell_{1}\gamma_{1}}\\&\quad+ x^{2\gamma_{2}} \Biggl(\sum _{\ell_{1}=1}^{\infty}\frac{\varGamma^{\ell _{1}-1}_{\gamma_{2}}(2)}{2^{\ell_{1}-1} \varGamma_{\gamma_{1}}(2\ell_{1})}t^{2\ell _{1}\gamma_{1}}+ \sum_{\ell_{1}=0}^{\infty}\frac{\varGamma^{\ell_{1}}_{\gamma_{2}}(2)}{2^{\ell _{1}} \varGamma_{\gamma_{1}}(2\ell_{1}+1)}t^{(2\ell_{1}+1)\gamma_{1}} \Biggr) \\ &\quad+ y^{2\gamma_{3}} \Biggl(\sum_{\substack{\ell_{1}=1}}^{\infty} \frac {\varGamma^{\ell_{1}-1}_{\gamma_{3}}(2)}{2^{\ell_{1}-1} \varGamma_{\gamma _{1}}(2\ell_{1})}t^{2\ell_{1}\gamma_{1}}+ \sum_{\substack{\ell_{1}=1}}^{\infty} \frac{\varGamma^{\ell_{1}}_{\gamma _{3}}(2)}{2^{\ell_{1}} \varGamma_{\gamma_{1}}(2\ell_{1}+1)}t^{(2\ell_{1}+1)\gamma _{1}} \Biggr) \\ &= z^{2} \bigl(E_{\gamma_{1}}\bigl(-t^{\gamma_{1}}\bigr)-1 \bigr)\\&\quad+x^{2\gamma_{2}} \biggl(\frac {2}{\varGamma_{\gamma_{2}}(2)} \biggl( \cosh_{\gamma_{1}} \biggl(\frac{\varGamma ^{0.5}_{\gamma_{2}}(2)}{2^{0.5}} t^{\gamma_{1}} \biggr)-1 \biggr)\\&\quad+ \biggl(\frac {2}{\varGamma_{\gamma_{2}}(2)} \biggr)^{0.5} \sinh_{\gamma_{1}} \biggl(\frac {\varGamma^{0.5}_{\gamma_{2}}(2)}{2^{0.5}} t^{\gamma_{1}} \biggr) \biggr) \\ &\quad+ y^{2\gamma_{3}} \biggl(\frac{2}{\varGamma_{\gamma_{3}}(2)} \biggl(\cosh _{\gamma_{1}} \biggl(\frac{\varGamma^{0.5}_{\gamma_{3}}(2)}{2^{0.5}} t^{\gamma _{1}} \biggr)-1 \biggr)\\&\quad+ \biggl(\frac{2}{\varGamma_{\gamma_{3}}(2)} \biggr)^{0.5} \sinh_{\gamma_{1}} \biggl(\frac{\varGamma^{0.5}_{\gamma _{3}}(2)}{2^{0.5}} t^{\gamma_{1}} \biggr) \biggr). \end{aligned} $$

As a particular case, if $\overline{\pmb{\gamma}}\rightarrow\overline {1}$, we obtain the solution of the wave-like equation in the integer 4D case:

3.21 $$ \begin{aligned}[b] \omega(t,x,y,z)& = z^{2} \bigl(e^{-t} -1 \bigr)+ \bigl(x^{2}+y^{2} \bigr) \bigl(\cosh(t)+\sinh(t)-1 \bigr) \\ & = z^{2}e^{-t}+ \bigl(x^{2}+y^{2} \bigr)e^{t}- \bigl(x^{2}+y^{2}+z^{2} \bigr). \end{aligned} $$

**Figure 3:**
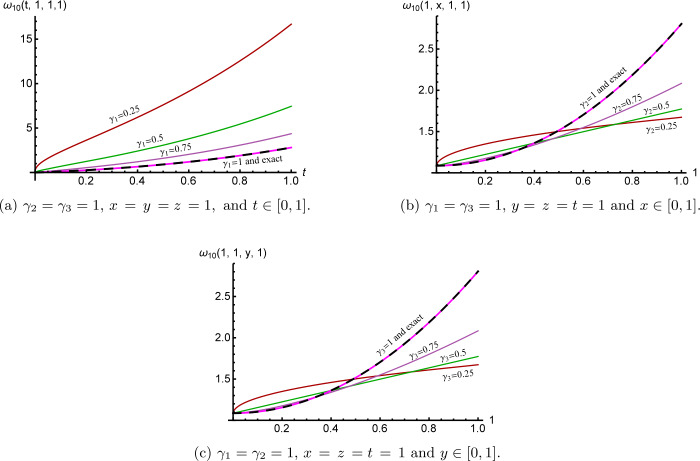
Cross-sections of the 10th approximate solution (3.20)

Figure 3 clarifies the cross-sections of the 10th approximate *γ̅*-Maclaurin solution (3.20) for several values of $\overline{\pmb{\gamma}} \in(0,1)^{3}$. Again, their performance shows that the *γ̅*-Maclaurin solution depends continuously on the fractional derivative parameters to attain the integer case solution, which in turn reflects some information about memory.

## Conclusion

In the current work, we provided an analytical simulation of the mutual impact for the existence of spatial and temporal memory indices in higher-dimensional PDEs in terms of *γ̅*-Maclaurin series, which is recently developed for the same purpose. Also, we presented a theoretical framework for the convergence of the *γ̅*-Maclaurin to support our idea. Practically, we employ an amendment of the power series technique to furnish analytically the solution of several well-known physical models with spatial and temporal memory indices together, namely the *γ̅*-heat and *γ̅*-wave-like models. The method exhibited a great potentiality in solving such hybrid models, and its performance is validated by comparing the projection of the obtained solutions with the available results in lower fractional spaces. Finally, the graphical analysis shows that the *γ̅*-Maclaurin solutions are homotopic mappings to attain the integer case solutions, which in turn may reflect some memory characteristics. For this reason, the Caputo fractional derivatives can be considered as memory indices.
